# Plain Radiographic Analysis of Laryngeal Dimensions in Young Children: Normal versus Croup

**DOI:** 10.3390/children9101532

**Published:** 2022-10-07

**Authors:** Youngdae Kim, Ji-Eun Park, Jung-Heon Kim

**Affiliations:** 1 Departments of Emergency Medicine, Ajou University School of Medicine, 164 World cup-ro, Yeongtong-gu, Suwon 16499, Korea; 2 Departments of Radiology, Ajou University School of Medicine, 164 World cup-ro, Yeongtong-gu, Suwon 16499, Korea

**Keywords:** cricoid cartilage, croup, glottis, larynx, radiographic film

## Abstract

(1) Background: Contrary to a tenet of the funnel-shaped pediatric larynx with the cricoid level being narrowest, recent studies show the glottis and subglottis as the narrowest levels. To locate the functionally narrowest level of the larynx, we reported normal laryngeal dimensions and their croup-related changes in young children. (2) Methods: We reviewed normal plain neck radiographs recorded for the evaluation of minor trauma or foreign bodies in 504 children aged ≤4 years who visited the emergency department from 2016 through 2021. Using computed tomography-based localization of the glottis, we radiographically defined the subglottis and cricoid. At these levels, we measured diameters and calculated cross-sectional areas (CSAs) on the radiographs. The values were compared to the equivalent values of a 1:1 age-matched population with croup. (3) Results: In the study population (n = 401), the narrowest diameter and CSA were observed in the glottis. In detail, the mean anteroposterior/transverse diameters were 9.8/3.4 mm at the glottis, 8.5/5.6 mm at the subglottis, and 7.4/6.8 mm at the cricoid (*p* < 0.001), respectively. In the same order, the mean CSAs were 26.5, 38.1, and 40.5 mm^2^ (*p* < 0.001). All dimensions were narrower in the croup population (*p* < 0.001). We found croup-related narrowing, namely reductions in the transverse diameter and CSA that were more severe closer to the glottis (*p* < 0.001), without differences per level in the anteroposterior diameter. (4) Conclusions: This study confirms the glottis as the narrowest level of the larynx in young children. In addition, level-based differences in croup-related narrowing suggest some point between the glottis and subglottis as the functionally narrowest level.

## 1. Introduction

According to autopsy-based anatomical knowledge, the larynx in young children is narrowest at the level of the cricoid cartilage, where it is circular in cross section and funnel-shaped [1]. However, since 2003, the glottis and subglottis have been suggested to be the narrowest laryngeal levels based on the findings of magnetic resonance imaging, bronchoscopy, or computed tomography (CT) [2,3,4,5]. Currently, the updated anatomy is debatable because some researchers opine that the rigid cricoid is prone to endotracheal tube (ETT)-induced injury, instead of the glottis, which is a part of the airway distensible by the elasticity of the vocal cords [6,7,8]. To assess the “functionally” narrowest level, it is ideal to study children who undergo endotracheal intubation, an infrequent procedure, in emergency departments (EDs). However, in intubated children, post-extubation stridor occurs in 1.2‒4.7% of cases [9,10]. This rarity of iatrogenic injury suggests a need for another entity to indirectly assess the laryngeal level most prone to injury.

The injury-prone level of the larynx may also be prone to infection because pressure and infection commonly cause laryngeal edema in the acute phase [11,12]. Croup results in edema of the conus elasticus, extending from the vocal cords to the cricoid cartilage [13]. Croup-induced hospitalization is associated with a decreased ratio of the narrowest laryngeal width to the upper tracheal width on plain radiographs [14]. In other words, the narrower the laryngeal diameter, the more severe the laryngeal obstruction. Hence, a comparison of croup-related narrowing among the laryngeal levels may indicate an obstruction-prone level that would be functionally, but not necessarily anatomically, narrowest. Plain radiography does not require sedation, vascular access, ionizing radiation, or radiocontrast, and is readily available in EDs despite its low resolution for delineating the airway–soft tissue interface. These strengths enable us to assess a larger, non-sedated population as compared to CT-based studies performed in 86‒130 sedated children [4,5].

The authors aimed to locate the functionally narrowest level of the pediatric larynx. Therefore, we reported laryngeal dimensions on plain radiographs in young children with normal larynx and compared the dimensions to the equivalent values in children with croup, a representative cause of upper airway obstruction.

## 2. Materials and Methods

### 2.1. Study Design, Setting, and Population

This retrospective study was conducted at the ED of a Korean academic hospital. The annual number of visits to the ED was approximately 21,000 and 10,000 children before and after the onset of the coronavirus disease 2019 pandemic, respectively. The study was approved by the institutional review board with a waiver for informed consent (IRB no. AJIRB-MED-MED-MDB-22-185).

We included children aged ≤4 years who underwent plain anteroposterior (AP) and lateral neck radiography for the evaluation of a minor trauma or foreign body, without a diagnosis related to allergy or infection, at the ED from 2016 through 2021. Exclusion criteria included swollen prevertebral soft tissue, overlapped soft tissue or guardian’s hands, extreme flexion or expiration, foreign bodies, torticollis, fracture, and indwelling devices. Hence, the study population was regarded to have normal laryngeal dimensions.

To compare the normal laryngeal dimensions to croup-related dimensions, children with moderate-to-severe croup (henceforth, “croup” unless stated otherwise) were age (year)-matched to the study population in a 1:1 ratio. Croup was defined as per the International Classification of Diseases, 10th Revision codes related to croup, as well as receipts of nebulized epinephrine and systemic dexamethasone or nebulized budesonide. Compared with mild croup, the moderate-to-severe croup was expected to ensure more frequent use of radiography and more discernible swelling on radiographs.

### 2.2. Definitions of Laryngeal Levels

We defined three levels, including the glottis, subglottis, and cricoid, within the larynx using CT-based localization of the glottis, a key landmark, according to the following process. In CT-based studies, the glottis was defined as the level that is the most cranial and teardrop-shaped, or is immediately caudal to the vocal cords [4,5,15]. In children undergoing both CT and radiography (1.7% (7 of the 401 children)), the glottis as defined above was indicated by a 3-dimensional cursor function (INFINITT PACS version 3.0.11.3 BN104; INFINITT Healthcare, Seoul, Korea) on CT scans, thus localizing the glottis on CT (Figure S1). This localization of the glottis was applied to radiographs of the same children, and the radiographically defined glottis was extrapolated to radiographs of the other children.

**Figure 1:**
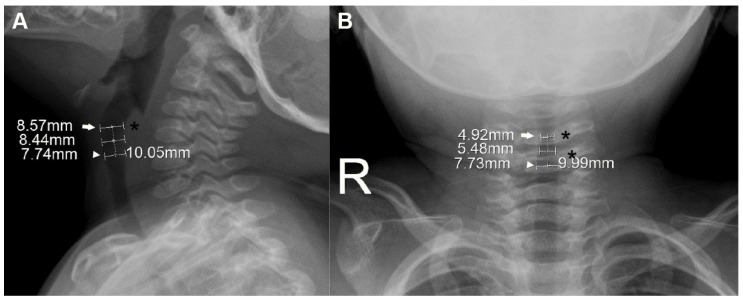
Measurement of laryngeal dimensions in a 15-month-old boy. On the lateral view (**A**), the glottis (arrow), which was extrapolated from computed tomography-based localization (see Figure S1), is further pointed out by the most protuberant portion of the posterior wall of the hypopharynx (asterisk). On the anteroposterior view (**B**), the glottis (arrow) is further specified as the narrowest level around the third and fourth cervical vertebrae (asterisks). The cricoid (arrowheads) was defined at a level 10.0 mm caudal to the glottis along the long axis (dotted lines). The subglottis was defined as the midpoint between the two levels. The measured anteroposterior (**A**) and transverse (**B**) diameters are noted at each level.

In the lateral view, the extrapolated glottis was further specified as the level of the most prominent portion of the posterior wall of the hypopharynx (Figure 1). This soft tissue covers the arytenoid cartilages, connects to the vocal cords, and is well demarcated from this view. From the AP view, the glottis was further specified as the narrowest level around the third and fourth cervical vertebrae, which is the location of the glottis in young children [13,16].

Based on the radiographically defined glottis, the cricoid and subglottis were defined on their radiographs by the following process. Given the radiolucency of cartilage, the cricoid was defined at a level 10.0 mm caudal to the glottis along the laryngeal long axis. This presumed distance between the glottis and the cricoid was modified from 8.4 mm, which was reported as the CT-based mean distance between the two levels in children aged <3 years [15]. To extrapolate this 8.4 mm to the slightly older population (age ≤4 years), we considered the possibly underestimated 1.5 mm in measuring the mean distance (i.e., the distance could be 9.9 mm), and a 48-month-old child’s calculated distance of 9.24 mm using the suggested formula (mean distance [mm] = 7.8 + 0.03 × corrected age [month]) [15]. The subglottis was defined as the midpoint between the two levels.

### 2.3. Measurement of Laryngeal Dimensions

At the three levels, the AP diameter on the lateral view and the transverse diameter on the AP view were independently measured in millimeters by two of the authors (YK and JHK) using electronic calipers at 100% magnified views with the zoom function of the INFINITT PACS (Figure 1). The final diameter was determined by the mean value of the two measurements and used to calculate the cross-sectional area (CSA) per level. Given the elliptical section of the larynx, the CSA was calculated using the following formula: CSA (mm^2^) = 3.14 × (0.5 × AP diameter) × (0.5 × transverse diameter). 

### 2.4. Comparison with Age-Matched Croup Population

In the croup population, the diameters were measured by one of the authors (JHK) and the CSAs were calculated as mentioned prior. Between a child in the study population and a matched child in the croup population, we calculated the percentages of croup-related narrowing in the AP diameter, transverse diameter, and CSA using the formula below. According to the dimensions at each level, this calculation was repeated 401 times at the patient-by-patient level:

$$\mathrm{Croup}-\mathrm{related}\;\mathrm{narrowing}\;(\%)=\;\frac{\mathrm{Dimension}\;\mathrm{of}\;\mathrm{child}\;A\;\left(\mathrm{study}\;\mathrm{population}\right)–\;\mathrm{Dimension}\;\mathrm{of}\;\mathrm{child}\;B\;\left(\mathrm{croup}\;\mathrm{population}\right)}{\mathrm{Dimension}\;\mathrm{of}\;\mathrm{child}\;A\;\left(\mathrm{study}\;\mathrm{population}\right)}\;\times 100$$

### 2.5. Statistical Analysis

Paired *t*-tests were performed to compare laryngeal dimensions between the glottis and subglottis, and between the subglottis and cricoid. Pearson’s correlation coefficients were used to assess correlations of age with the dimensions. Bland–Altman plots as unit differences were created to quantify the agreement between the two independent measurements, with a limit of the maximum acceptable difference of 1.0 mm defined a priori. Student’s *t*-tests were used to compare the dimensions of the study population and of the age-matched croup population. The percentages of croup-related narrowing per dimension were compared between the glottis and subglottis, and between the subglottis and cricoid using Wilcoxon signed-rank tests. This comparison indicates a level-based difference in croup-related narrowing. A *p* < 0.05 was considered significant. We used the MedCalc Statistical Software version 20.109 (MedCalc Software Ltd., Ostend, Belgium).

## 3. Results

### 3.1. Baseline Characteristics

**Figure 2:**
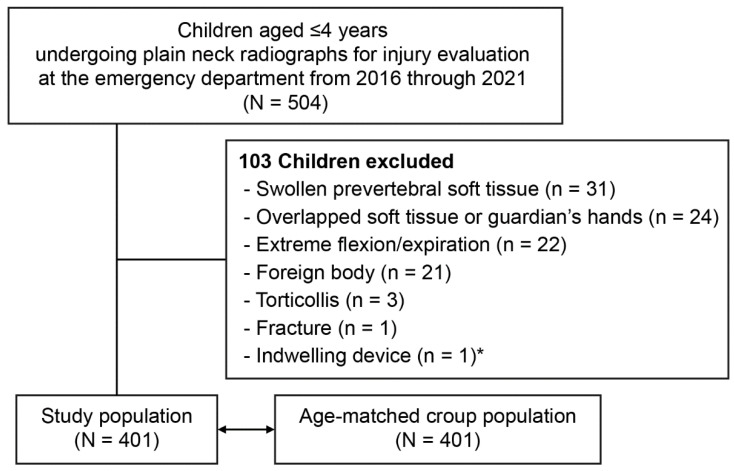
Flowchart for the study population. * Ventriculoperitoneal shunt.

Of the 504 children aged ≤4 years who underwent the radiography, 401 were analyzed (Figure 2). The study population had a median age of 26.0 months (interquartile range, 14.8–38.0), included 168 girls (41.9%), and had a mean weight of 12.5 ± 2.9 kg. The median age and mean weight showed no differences compared with those values of the age-matched croup population (vs. 26.0 months [15.0–37.0]; *p* = 0.937) (vs. 12.9 ± 3.0 kg; *p* = 0.062).

### 3.2. Measurement of Laryngeal Dimensions

**Figure 3:**
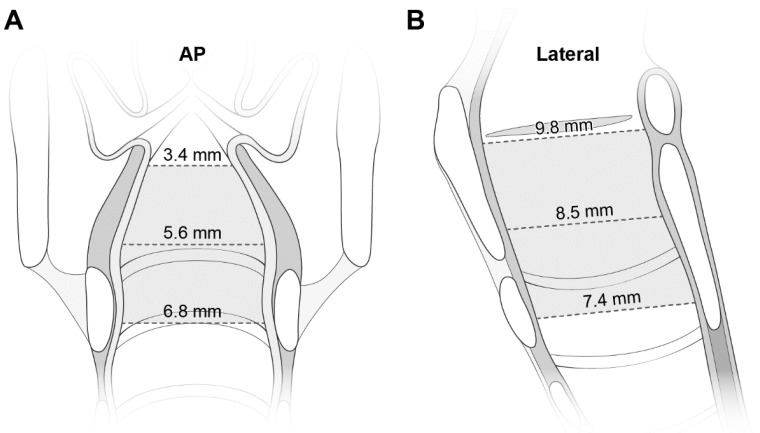
The laryngeal configuration based on the measured transverse diameters on AP views (**A**) and AP (i.e., longitudinal) diameters on lateral views (**B**). The diameters are noted at each level. See more numerical data in Table 1. AP indicates anteroposterior.

Table 1 lists the dimensions at the three levels. The mean values of the transverse diameter and CSA were smallest at the glottis, and increased caudally. Despite the reverse direction of the increase in the AP diameter, the increase was smaller than that in the transverse diameter (2.4 mm vs. 3.4 mm). All diameters were correlated with age (Figure S2). Figure 3 shows the laryngeal configuration based on these measurements.

The Bland–Altman plots showed that all biases were within the limit of the maximum acceptable difference (Figure S3). The glottic transverse diameter had the highest intraclass correlation coefficient among all diameters (Table S1).

### 3.3. Comparison with Age-Matched Croup Population

All dimensions were narrower in the croup population (Table 2). The mean CSAs in the croup population were 40.4%, 46.5%, and 56.5% of the equivalent values in the study population for the glottis, subglottis, and cricoid, respectively. As for the level-based difference in croup-related narrowing, we found greater degrees of narrowing in the upper levels within the larynx. In detail, the transverse diameter and CSA showed more severe reduction the closer we moved to the glottis (*p* < 0.001; Table 3). In contrast, no such a difference was noted in the AP diameter. The AP diameter showed a smaller difference in the mean values of the narrowing between at the glottis and at the cricoid, compared with the transverse diameter and CSA (AP, 2.6% vs. transverse, 22.6% and CSA, 17.1%).

## 4. Discussion

This study confirms that the glottis is the narrowest laryngeal level on plain radiographs of non-sedated children aged ≤4 years. In addition, the croup-related narrowing being more severe at the glottis and subglottis than at the cricoid suggests that some point between the upper two levels is more prone to swelling upon infection than the other level within the larynx. Our findings strengthen the recent updates in laryngeal anatomy [2,3,4,5] and indicate an obstruction-prone level. The findings will aid in pediatric airway management.

This study is consistent with the recent studies showing that the pediatric larynx is narrowest at the glottis and elliptical in section, refuting the old tenet of a funnel-shaped larynx [2,3,4,5]. For instance, the shortest dimension was the glottic transverse diameter (Table 1). In accordance with a CT-based study [5], ours showed the transition of the axial section from an ellipse to a near-circle as we progress caudally (see ratios in Table 1). In a recent study on radiographic findings of croup, the mean value of the narrowest laryngeal width was 2.0 mm in 192 children (mean age, 2.1 years) [14]. This value approaches the mean glottic transverse diameter of 1.8 mm in our croup population (mean age, 2.2 years) (Table 2).

This study has implications for updating the knowledge on laryngeal anatomy and possibly, for predicting the location prone to obstruction represented by croup. The old preference for uncuffed ETTs in pediatric intubation is predicated on a funnel-shaped larynx and the myth of uncuffed ETTs fitting at the cricoid level. The false configuration-based myth has been proved wrong by the recently updated anatomy and by this study [2,3,4,5]. The croup-related narrowing centered on the glottis and subglottis corresponds with the optical coherence tomography-proven correlation of intubation duration with wall thickness in the glottis and subglottis, not with that in the upper trachea [17]. This analogy suggests a common acute pathology between ETT-induced injury and croup, and supports the glottis and subglottis as the functionally narrowest level, against the opinion by researchers supporting the cricoid as the functionally narrowest level [6,7,8]. With additional evidence, our findings could lead to a prediction of the laryngeal level prone to obstruction by ETTs or infections in emergency settings.

We speculate that our finding of the obstruction-prone level remains valid even after consideration of potential errors in measuring the glottic transverse diameter. At this level, the mean transverse diameter of 3.4 mm is shorter than the reported values ranging from 5.3 to 7.5 mm [4,18]. This gap indicates an underestimation of the diameter by the overlapped soft tissue [14] or by the adducted vocal cords during phonation, such as crying. Although the former error is inherent in the uniplanar AP view, we see at least the narrowing tendency going cephalad. The latter error can be remarkable in young children who breathe rapidly and are poorly cooperative while recording radiographs. In patients aged <18 years, the ultrasonography-measured mean distance between the abducted vocal cords is approximately 4 mm [19], which indicates the glottic transverse diameter during respiration. The value approximates our 3.4 mm and is shorter than the transverse diameters at the other levels (5.6–6.8 mm; Table 1). As shown in Table S1, the highest intraclass correlation coefficient of the glottic transverse diameter means the best agreement between the two measurements. The abovementioned errors can be circumvented by measuring the subglottic diameter, which is better demarcated on radiographs, and is used interchangeably with the glottic diameter [4,5,15]. At the glottis, it is prudent to recognize merely a narrowing per se on radiographs, instead of its precision on a millimeter basis.

This study has limitations. First, even after excluding the 22 children for their extreme cervical flexion or expiration while taking radiographs, we admitted some degree of flexion, expiration, or rotation given the inherent lack of cooperation among young children. This tendency might have been intensified by the children with severe croup who may have been restless due to their symptoms. Second, although the presumed position of the cricoid might affect the measurements, this flaw might be unproblematic considering the biases ranging from −0.1 to 0.2 with excellent agreement between the two measurements of the cricoid diameters (Figure S3 and Table S1). Third, croup-related narrowing is not applicable to mild croup because of the operational definition of croup and recommendations against the routine use of radiography. Finally, despite the variable growth levels of the children, weight was not considered for matching. However, the mean weight did not differ between the study and croup populations.

## 5. Conclusions

Recently, our knowledge on laryngeal anatomy in young children has changed such that the larynx features the glottis as the anatomically narrowest level, and some point between the glottis and subglottis as the functionally narrowest level. These findings strengthen the recent imaging-based updates in laryngeal anatomy and support the upper two levels in the midst of the debate on the functionally narrowest levels. With more evidence, this study can guide pediatricians or emergency physicians in predicting the site of obstruction by infection or intubation in the larynx.

## Figures and Tables

**Table 1:** Laryngeal dimensions per level (n = 401).

Dimension	Glottis	Subglottis	Cricoid	*p* *
AP diameter, mm	9.8 ± 1.5	8.5 ± 1.4	7.4 ± 1.5	<0.001
Transverse diameter, mm	3.4 ± 1.8	5.6 ± 1.0	6.8 ± 1.0	<0.001
AP-to-transverse ratio ^†^	2.9:1	1.5:1	1.1:1	NA
CSA, mm^2^	26.5 ± 15.1	38.1 ± 11.2	40.5 ± 12.5	<0.001

The values are expressed as the means ± standard deviations, except the ratios. * Common *p* values of paired *t*-tests: glottis vs. subglottis and subglottis vs. cricoid. ^†^ Calculated using the mean diameters. AP indicates anteroposterior; CSA, cross-sectional area.

**Table 2:** Comparison with the age-matched croup population.

Dimension	Study Population (n = 401)	Croup Population (n = 401)	*p* *
Glottis			
AP diameter, mm	9.8 ± 1.5	7.1 ± 1.6	<0.001
Transverse diameter, mm	3.4 ± 1.8	1.8 ± 1.2	<0.001
CSA, mm^2^ *	26.5 ± 15.1	10.7 ± 8.5	<0.001
Subglottis			
AP diameter, mm	8.5 ± 1.4	6.3 ± 1.6	<0.001
Transverse diameter, mm	5.6 ± 1.0	3.4 ± 1.1	<0.001
CSA, mm^2^^†^	38.1 ± 11.2	17.7 ± 8.9	<0.001
Cricoid			
AP diameter, mm	7.4 ± 1.5	5.5 ± 1.5	<0.001
Transverse diameter, mm	6.8 ± 1.0	5.0 ± 1.3	<0.001
CSA, mm^2^ *	40.5 ± 12.5	22.9 ± 11.0	<0.001

The values are expressed as the means ± standard deviations. * Student’s *t*-test. ^†^ Mean values of the calculated CSAs. AP indicates anteroposterior; CSA, cross-sectional area.

**Table 3:** Level-based differences in the percentages of croup-related narrowing.

Narrowing, %	Glottis	Subglottis	Cricoid	*p*
AP diameter	28.2 (14.3–38.2)	25.1 (11.9–37.8)	25.6 (12.9–38.0)	NS *
Transverse diameter	50.0 (21.4–68.6)	41.4 (26.9–52.6)	27.4 (14.7–39.8)	<0.001 ^†^
CSA	63.2 (42.7–79.3)	56.6 (38.5–70.4)	46.1 (29.5–60.0)	<0.001 ^†^

The values are expressed as the medians (interquartile ranges) of the percentages representing the narrowing. * The *p* values of the Wilcoxon signed-rank tests were 0.089 (glottis vs. subglottis) and 0.932 (subglottis vs. cricoid). ^†^ Common *p* values of Wilcoxon signed-rank tests: glottis vs. subglottis and subglottis vs. cricoid. AP indicates anteroposterior; CSA, cross-sectional area; NS, not significant.

## Data Availability

The datasets analyzed during the current study are not publicly available due to the Korean Bioethics and Biosafety Act, but may be available from the corresponding author upon reasonable request.

## Supplementary Materials

The following materials are available online at https://www.mdpi.com/article/10.3390/children9101532/s1. Figure S1: Computed tomography-based landmarks. The glottis was defined at the level indicated by 3-dimensional cursors (solid lines) on axial (A), sagittal (B), and coronal (C) scans. Figure S2: Correlations of age with the diameters. Correlation coefficients are noted on the top of each thumbnail (p < 0.001). AP indicates anteroposterior. Figure S3: Bland–Altman plots of the AP and transverse diameters per level. All biases are within the limit of the maximum acceptable difference defined a priori (solid lines above and below another solid line for the biases). See details in Table S1. AP indicates anteroposterior. Table S1: Details of Bland–Altman plots with ICCs (See plots in Figure S3). AP indicates anteroposterior; LOA, limit of agreement; ICC, intraclass correlation coefficient; CI, confidence interval.
