# Supplementary material for: Plain Radiographic Analysis of Laryngeal Dimensions in Young Children: Normal versus Croup
Source: Children (Basel). 2022 Oct 7;9(10):1532. doi: 10.3390/children9101532 (PMC9600057; doi:10.3390/children9101532)
Supplement: Supplementary file 1 [file children-09-01532-s001.zip › Table_S1.pdf]

Table S1 Details of Bland-Altman plots with ICCs (See plots in Figure S3)

Dimension	Bias, mm	LOAs, mm	ICC (95% CIs)
Glottis			
AP diameter	−1.0	−3.6 to 1.5	0.823 (0.785 to 0.855)
Transverse diameter	−0.2	−2.1 to 1.6	0.932 (0.917 to 0.944)
Subglottis			
AP diameter	−0.1	−1.6 to 1.5	0.917 (0.899 to 0.932)
Transverse diameter	−0.1	−1.7 to 1.5	0.828 (0.791 to 0.859)
Cricoid			
AP diameter	0.2	−1.4 to 1.8	0.924 (0.908 to 0.938)
Transverse diameter	−0.1	−1.6 to 1.4	0.866 (0.837 to 0.890)

AP indicates anteroposterior; LOA, limit of agreement; ICC, intraclass correlation coefficient; CI, confidence interval.
