# Supplementary figures and images for: Plain Radiographic Analysis of Laryngeal Dimensions in Young Children: Normal versus Croup
Source: Children (Basel). 2022 Oct 7;9(10):1532. doi: 10.3390/children9101532 (PMC9600057; doi:10.3390/children9101532)

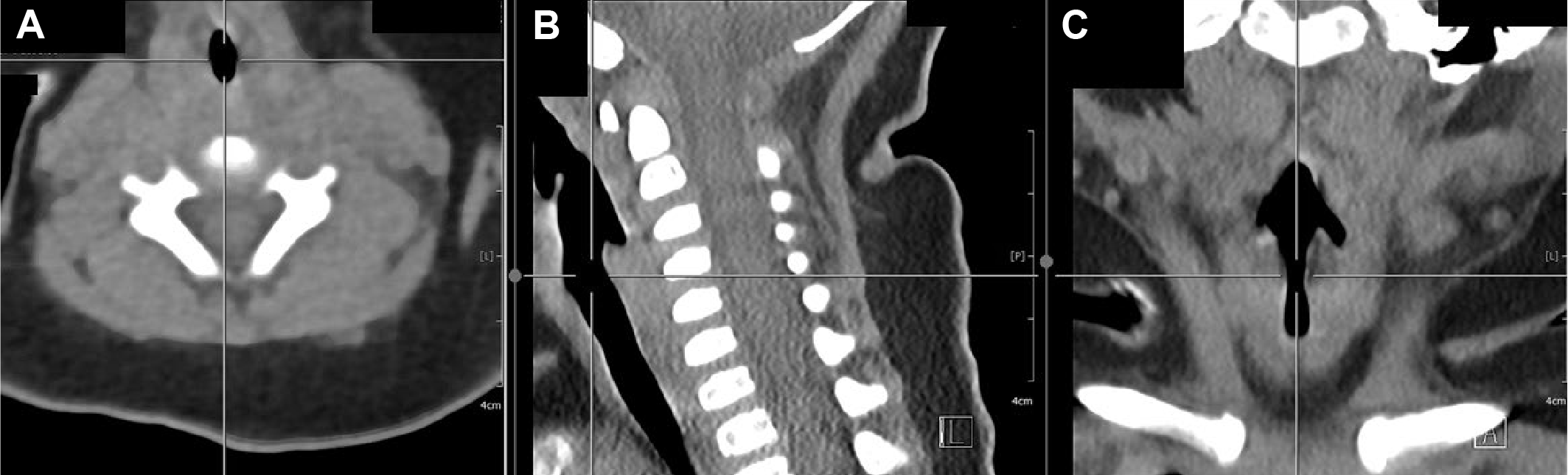

Supplement: Supplementary file 1 [file children-09-01532-s001.zip › Figure_S1.tif]

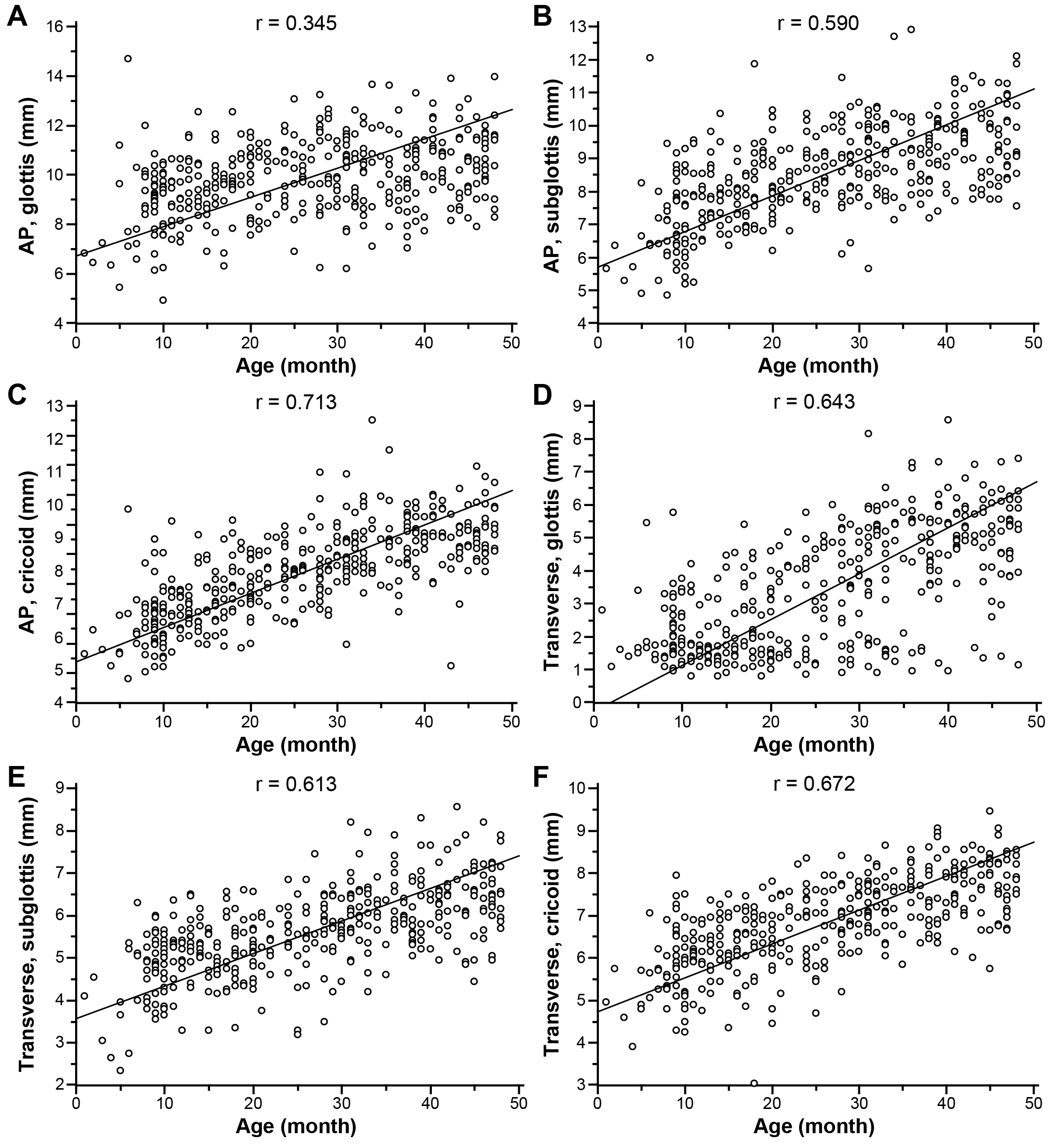

Supplement: Supplementary file 1 [file children-09-01532-s001.zip › Figure_S2.tif]

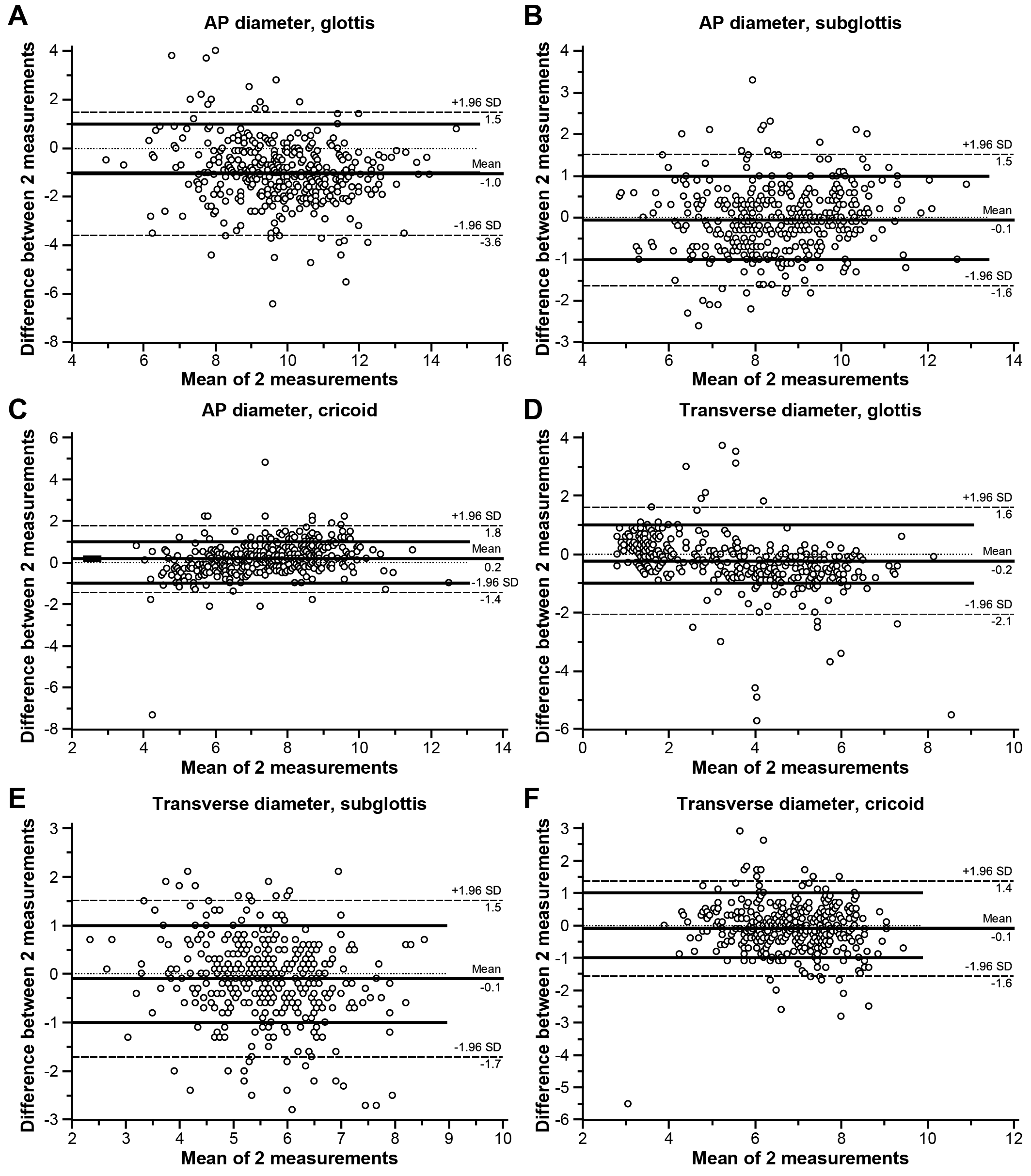

Supplement: Supplementary file 1 [file children-09-01532-s001.zip › Figure_S3.tif]
